# Prediction of discoid lateral meniscus based on tibial plateau anatomical factors and machine learning

**DOI:** 10.3389/fmed.2026.1814295

**Published:** 2026-09-28

**Authors:** Xixin Jin, Haoyu Wang, Zelong Yang, Yongtao Mao, Xujin Wu

**Affiliations:** 1 Department of Orthopedics, The Third People's Hospital of Bengbu, Bengbu, Anhui, China; 2 Department of Orthopedics, The Second Affiliated Hospital of Soochow University, Suzhou, Jiangsu, China

**Keywords:** clinical prediction model, dscoid lateral meniscus, machine learning, radiographic parameters, tibial plateau morphology

## Abstract

**Objectives:**

The discoid lateral meniscus (DLM) is the most prevalent variant type of the lateral meniscus, and its formation is closely related to specific anatomical features of the tibial plateau. The objective of this study was to systematically evaluate the combined predictive contribution of multiple tibial plateau anatomical parameters and to develop subtype-specific machine-learning models using routinely obtainable radiographic features for the identification of both CDLM and the comparatively understudied ICDLM.

**Methods:**

This was a retrospective clinical study, and a total of 494 patients were included, of which the experimental group consisted of 145 patients with complete discoid lateral meniscus (CDLM) and 64 patients with incomplete discoid lateral meniscus (ICDLM), and the control group consisted of 285 patients with normal lateral meniscus (NLM). A number of tibial plateau anatomical parameters were measured radiographically, including tibial plateau width, fibular head height, lateral joint space, height of lateral tibial spine, height of medial tibial spine, lateral slope of the lateral tibial spine, lateral slope of the medial tibial spine, tibial spinous width and tibial eminence width. All data were analyzed by gender grouping in order to compare differences between and within groups. Subsequently, the Least Absolute Shrinkage with Selection Operator (LASSO) regression algorithm was used to screen the feature variables. Seven machine learning prediction models (including: Random Forest, XGBoost, Support Vector Machine (SVM), Logistic Regression, K-Nearest Neighbor (KNN), Naive Bayes, and Gradient Boosting) were constructed based on the screened feature variables, and the best model was selected. Using SHAP (Shapley Additive Explanations) to quantitatively analyze the contribution of each characteristic variable, thereby assessing the relative importance of the characteristic factors and increasing the interpretability of the model.

**Results:**

Gender, lateral joint space, height of lateral tibial spine, lateral slope of the lateral tibial spine, lateral slope of the medial tibial spine, and tibial eminence width/tibial plateau width can be used as characteristic parameters of lateral discoid meniscus and lateral incomplete discoid meniscus. Based on these parameters, the best-performing models achieved good discriminative performance for both CDLM and ICDLM in the held-out validation sets, with AUC values of 0.885 and 0.861, respectively.

**Conclusion:**

Tibial plateau anatomical parameters may facilitate the identification of DLM. Machine-learning models integrating these parameters provide an efficient radiograph-based approach to support the early recognition of DLM.

## 1 Introduction

The knee meniscus is an important component of the knee joint and plays a major role in the biomechanics of the knee in distributing load and protecting the articular cartilage (1). The discoid lateral meniscus (DLM) is the most common congenital variant of the lateral meniscus often found in childhood and adolescence, first reported by Young (2). Racial and regional disparities exist in DLM, which is more prevalent in Asian populations (10.9%–16.6%) and common in females (69.6%) (3–5). In up to 79%–97% of cases, DLM is often found to be present on both sides (6). In terms of morphology, the DLM is not as semilunar as the NLM, but is instead disc-shaped and covers over 70% of the lateral tibial plateau (7). Histologically, the collagen fibers of the DLM ring are sparse and disorganized (8–12). From a clinical perspective, DLM predisposes patients to underlying peripheral rim instability, lateral femoral condylar dysplasia, and abnormal axial alignment of the lower extremities (12, 13). These histomorphometric features of the DLM make it more susceptible to tearing than the NLM, often manifesting as discomfort such as knee pain, swelling, popping, locking, and limited motion (14, 15). Moreover, as the disease progresses, patients with DLM injuries often suffer from a combination of cartilage damage to the knee, and in some cases, severe knee deformities and osteoarthritis of the knee can occur (16–18). Therefore, a quick and simple diagnosis of a discoid meniscus is more helpful in preventing meniscal damage and reducing the incidence of osteoarthritis in the knee joint.

Currently, the Watanabe method is most often used to classify DLMs (19). Based on the area covered by the tibial plateau of the meniscus, the DLM is classified as complete discoid meniscus (CDLM), incomplete discoid meniscus (ICDLM), Wrisberg meniscus. Among them, complete DLM and incomplete DLM were similar in organization, and the differences were mainly in the coverage of the lateral tibial plateau in complete DLM than in incomplete DLM. Wrisberg's meniscus Morphologically and histologically similar to the NLM, it is mainly characterized by the attachment of the posterior horn of the lateral meniscus to the medial femoral condyle through the Wrisberg's ligament, with a low prevalence of only 0.2% (20). Due to significant morphological and histological differences between the Wrisberg and discoid menisci and DLM, the Wrisberg meniscus was excluded from this study. Only complete and incomplete DLM were included.

There are a variety of ways to diagnose DLM, including arthroscopy, imaging and physical examination.

Arthroscopy is considered the reference standard for the definitive diagnosis and morphological classification of DLM and enables direct assessment of associated meniscal tears and peripheral rim stability (21–23). Based on physical examination, the lateral discoid meniscus is prone to knee strangulation, popping, and other uncomfortable symptoms, but the symptoms are similar to those of a common meniscus injury, so it is not used to confirm the diagnosis of DLM. The imaging tests mainly include magnetic resonance imaging (MRI), x-rays of the knee and ultrasound scans of the knee. Among these, MRI is the most commonly used imaging modality for diagnosing DLM, which is mainly characterized by the number of consecutive connected consecutive sagittal planes of the anterior and posterior corners of the lateral meniscus ≥3 (24). However, the shortcomings of MRI, such as time-consuming, costly, and low prevalence, have prevented it from being used for the extensive examination of discoid menisci, and it is even more difficult to perform MRI on patients with claustrophobia (25). Furthermore, MRI remains a subject of debate when it comes to the examination of discoid menisci in paediatric and elderly populations, which are known to be susceptible to growth and ageing (26). X-rays are widely used because of their high popularity, convenient examination and low cost. Some studies have reported that the morphology of DLM can be diagnosed by the unique features of the skeletal structure of the knee on x-ray. In patients diagnosed with DLM, the skeletal structure of the knee is abnormal, especially the femoral condyles and the tibial plateau. Diagnosis of DLM can be made on the basis of parameters such as the height of the fibular head, the condition of the lateral joint space, and the slope of the lateral tibial plateau. However, the prediction and accuracy based on a single indicator are not optimal (27–32). Furthermore, standing full-length films of the lower extremity of the knee can be utilised to assess the axial alignment of the lower extremity of the knee and provide a reference for the treatment and prognosis of DLM. X-rays are therefore more conducive to healthy screening and initial diagnosis of disease and play an important role in preventing discoid meniscus injuries.

Artificial intelligence (AI) technology refers to the use of computer algorithms to simulate, extend and expand human perceptual, learning and operational capabilities. In recent years, advanced AI technologies have been used in a wide range of fields, including physics, chemistry and medicine. Machine Learning (ML) algorithms, as an important branch in the field of Artificial Intelligence, are used to construct predictive models for a target task by dividing a data set into a training set and a test set, and constructing predictive models for the target task from the training data (33). In contradistinction to conventional statistical methodologies, which presuppose linear relationships between predictive features and outputs, machine learning algorithms [including decision tree-based algorithms and support vector machines (SVMs) with nonlinear kernel functions] possess the capacity to capture nonlinear relationships between features. Consequently, they are able to construct more accurate predictive models (34). Consequently, machine-learning-based predictive models frequently demonstrate enhanced accuracy and adaptability when confronted with complex outcomes exhibiting multivariate effects.

Numerous studies have demonstrated significant differences in the characterization of bone structure in DLM knee radiographs compared to NLM. However, the diagnostic outcome of DLM is the result of a combination of multiple anatomical factors of the knee. And to date, no studies have systematically explored the complex interrelationships between these factors and the extent to which they influence DLM. Furthermore, prediction models for single factors are typically characterized by a reduced level of accuracy, with a prediction accuracy of approximately 50% (29–31). In addition to this, most studies have focused on the characteristics of CDLM, ignoring the effects of ICDLM. Therefore, this study will build on previous studies to further explore the influence of several new knee tibial plateau anatomical factors on DLM. And for the first time, incomplete discoid meniscus was included in the framework of the analysis. In contradistinction to the conventional single-variable prediction model, this study utilized a machine learning approach to construct a prediction model by combining multiple variables, with the objective of enhancing prediction accuracy.

Therefore, the aims of this study were (1): to identify and screen the anatomical factors of the tibial plateau for lateral discoid meniscus of the knee; and (2): to construct a clinical prediction model for lateral discoid meniscus of the knee based on a machine learning method.

## 2 Materials and methods

### 2.1 Patients and study design

This study comprised 494 patients with lateral meniscus who attended the Second Affiliated Hospital of Soochow University from January 2019 to June 2025. Of these patients, 145 had CDLM and 64 had ICDLM. The remaining 285 patients with NLM were included as a control group (Because this retrospective study included all eligible patients available during the predefined study period, no *a priori* sample size calculation was performed. As a pragmatic assessment of the control sample, 17 candidate predictor parameters were considered before LASSO selection. With 70% of the cohort allocated to model training, at least 243 NLM controls were required to provide approximately 10 non-event observations per candidate predictor in the training set (17 × 10/0.70). The 285 NLM controls included in this study exceeded this pragmatic threshold). The inclusion criteria of the patients were (1) patients whose diagnosis was confirmed by orthopedic specialists and imaging specialists. The diagnosis and classification of DLM were initially based on predefined MRI morphological criteria. All 209 patients with MRI-diagnosed DLM subsequently underwent arthroscopic evaluation and treatment, and the arthroscopic findings were used to confirm the diagnosis and subtype classification. The NLM control group comprised individuals whose lateral menisci were assessed as normal on MRI obtained during routine health examinations. Arthroscopic verification was not performed in the control group because there was no clinical indication to expose individuals with normal MRI findings to an invasive procedure; (2) having complete clinical and imaging data. The exclusion criteria were: (1) the presence of knee fractures and fracture history; (2) suffering from inflammatory osteoarthritis of the knee joint, and the bony structure of the knee joint has been significantly affected; (3) combined with ligament injuries, such as anterior cruciate ligament, posterior cruciate ligament and medial/lateral collateral ligament injuries; (4) incomplete skeletal development, including epiphyseal closure; (5) the patients' basic data and imaging data were missing or incomplete; (6) Accompanied by other serious systemic diseases.

All operations in this study followed the principles of the 1964 Declaration of Helsinki, and studies involving human subjects were approved and authorized by the Ethics Committee of the Second Affiliated Hospital of Soochow University (No. JD-HG 2024-088). This study was an anonymous retrospective study and data analysis, and informed consent was obtained from patients for all data acquisition.

### 2.2 Data collection

For the patients included in this study, the following indicators were retrospectively collected for this study:

1. Baseline characteristics of the patients: gender, age, height, weight, and body mass index (Body Mass Index BMI) of the patients.
2. The dependent variable in this study is the morphology of the patient's lateral meniscus. The diagnostic criteria for complete discoid lateral meniscus are as follows: Magnetic resonance imaging (MRI) shows: In the coronal plane, the meniscus appears as a broad, low-signal structure that completely covers the lateral tibial plateau and extends into the intercondylar fossa, losing its normal triangular configuration. The minimum width of the meniscus body is ≥15 mm. In the sagittal view: The “bow tie sign” is significantly elongated, with a transverse, band-like, low signal intensity visible in four or more consecutive slices. Diagnosis of incomplete discoid lateral meniscus involves the following MRI findings: Coronal view: Marked widening of the meniscal body (appearing as a broad triangle/trapezoid), covering >50% but <100% of the lateral tibial plateau width without reaching the intercondylar fossa. Sagittal view: Elongated “bow tie sign” with transverse band-like low signal intensity visible across three consecutive slices. Then get a more accurate classification through further arthroscopic examination.
3. The imaging parameters are as follows: All patients underwent standardized knee radiography, in which the knee was fully extended in a neutral position and positioned in close proximity to the detector, with the detector placed at a distance of 1.0 meters from the radiation source. The patella was oriented anteriorly in order to eliminate the effects of knee rotation and flexion on measurement results. The x-ray tube was angled at an angle of 10–15° to the head-to-tail direction, thereby ensuring the production of a complete intercondylar spine (27). All imaging views for patients included in the study were acquired at our hospital's imaging center using standardized procedures by senior radiologists from our institution. All angle and distance measurements were performed using the standard imaging software long employed by our institute. All measurements were conducted under the guidance of senior radiology specialists. Two trained orthopedic surgeons independently performed two rounds of measurements at different time points. Intraobserver and interobserver reliability was evaluated using intraclass correlation coefficients (ICCs) with 95% confidence intervals (CIs). An ICC greater than 0.75 was considered indicative of acceptable measurement reliability. Both observers were blinded to the MRI-based diagnosis and clinical group assignment during all radiographic measurements. The following exposition will methodically introduce the relevant radiographic parameters on standard knee x-rays.

Tibial plateau width (TW): The maximum distance along the line connecting the medial and lateral margins of the tibial plateau on x-ray (Figure 1a);

**Figure 1:**
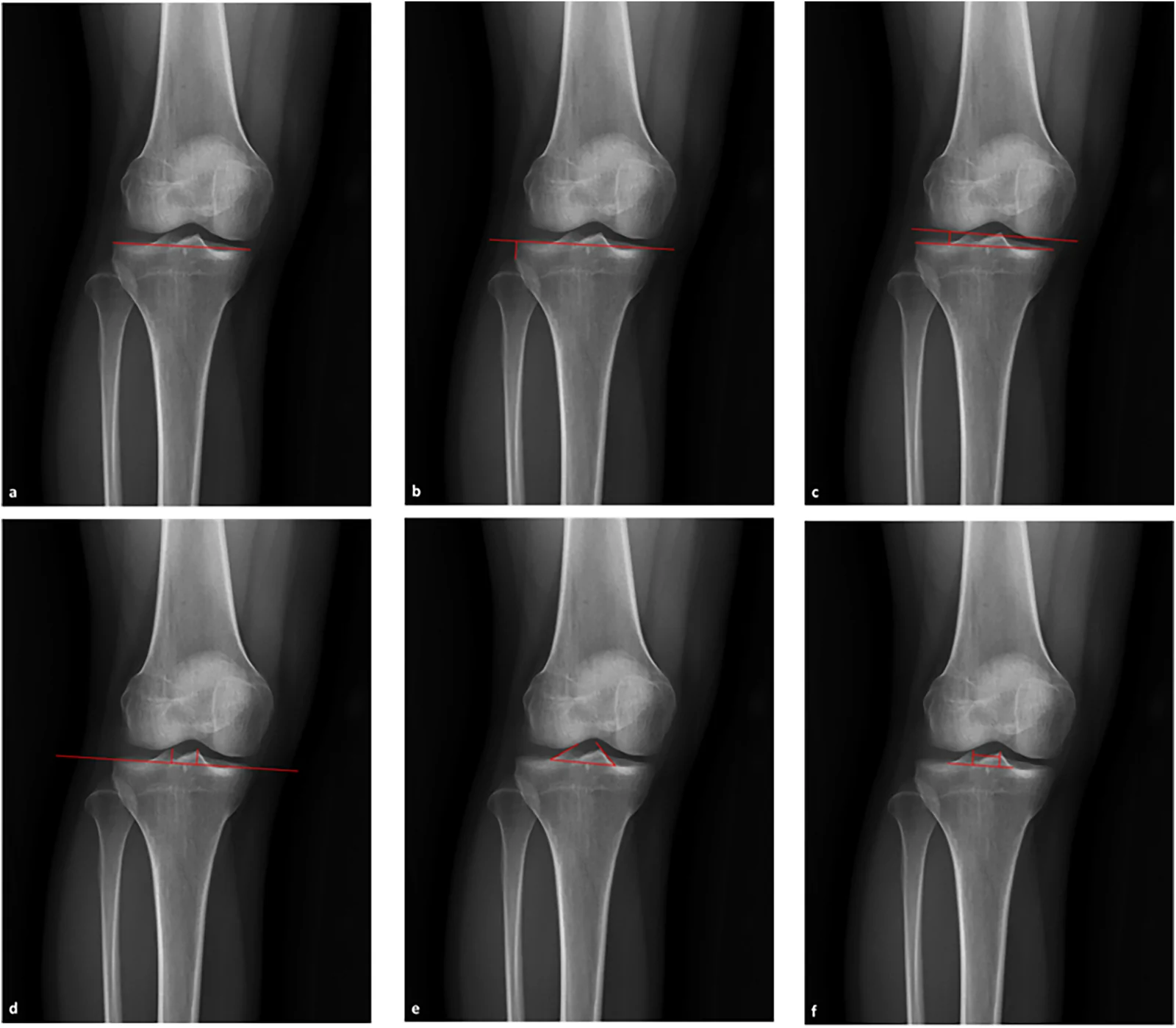
Measurement of relevant imaging parameters on standard knee x-rays: **(a)** Tibial plateau width (TW); **(b)** Fibular height (FH); **(c)** Lateral joint space (LJS); **(d)** Height of lateral tibial spine (HLTS) and Height of medial tibial spine (HMTS); **(e)** Lateral slope of the lateral tibial spine (LSLTS) and Lateral slope of the medial tibial spine (LSMTS); **(f)** Tibial eminence width (TEW) and Tibial spinous width (TSW).

Fibular height (FH): The vertical distance between the highest point of the fibular head and the horizontal plane of the tibial plateau (Figure 1b);

Lateral joint space (LJS): Distance between the tangent lines of the lateral femoral condyle and the lateral tibial condyle (Figure 1c);

Height of lateral tibial spine (HLTS) and Height of medial tibial spine (HMTS): The vertical distance between the horizontal line connecting the tibial plateau and the highest point of the lateral/medial tibial spine (Figure 1d);

Lateral slope of the lateral tibial spine (LSLTS) and Lateral slope of the medial tibial spine (LSMTS): The angle formed between the tibial joint line and the line drawn along the lateral aspect of the lateral/medial tibial tuberosity (Figure 1e);

Tibial eminence width (TEW): The distance between the tips of the lateral and medial tibial spines (Figure 1f);

Tibial spinous width (TSW): The horizontal distance between the points where the lateral tibial spine begins to slope and where the medial tibial spine begins to slope (Figure 1f).

Additionally, to account for the influence of tibial plateau width across different populations on spinous process width, this study calculated the ratios of the distance between the tibial eminence width (TEW) and the tibial spinous width (TSW) to the tibial plateau width (TW). It also introduced a new variable: the angular difference in lateral inclination between the medial and lateral spinous processes.

### 2.3 Statistical analysis

Continuous variables were assessed for normality using the Shapiro–Wilk test and for homogeneity of variance using Levene's test. Normally distributed variables were compared among the three groups using Single-factor analysis of variance (ANOVA) (35); otherwise, the Kruskal–Wallis test was used. Categorical variables, including gender and meniscus position, were compared using the chi-square test or Fisher's exact test, as appropriate. All tests were two-sided, and *p* < 0.05 was considered statistically significant. Given the hypothesis-generating nature of the comparisons across multiple radiographic parameters, these analyses were interpreted as exploratory.

### 2.4 Machine learning model development

Before model development, records with missing or incomplete clinical or imaging data had already been excluded during cohort assembly; therefore, no imputation was performed. Gender was treated as a categorical predictor, and continuous predictors were standardized using the mean and standard deviation estimated from the training data. The resulting transformations were applied unchanged to the validation data. This study employed the Least Absolute Shrinkage and Selection Operator (LASSO) regression to perform feature selection on data. LASSO regression is an extension of linear regression algorithms, primarily applied to feature selection and model optimization in high-dimensional data (36–38). To minimize information leakage, data splitting preceded all preprocessing and feature selection. LASSO regression was performed within the training set using candidate predictors. The penalty parameter was selected by 10-fold cross-validation at the value associated with the minimum cross-validated loss, yielding predictors with non-zero coefficients. This feature set was subsequently applied without modification to the validation data.

This study proposes a novel predictive model for the lateral meniscus, constructed on the basis of a comprehensive set of characteristic parameters. The data was randomly split into training and validation sets at a 7:3 ratio. Seven machine learning models were employed to construct predictive models, including: Random Forest, XGBoost, Support Vector Machine (SVM), Logistic Regression, K-Nearest Neighbors (KNN), Naive Bayes, and Gradient Boosting. The seven algorithms were trained using the same predictor feature set. Hyperparameters were optimized with Hyperopt within 10-fold cross-validation in the training set. Each model was subsequently refitted using the complete training set and its optimal hyperparameter combination, and performance was assessed once in the held-out validation set. This study comprehensively evaluated model performance by calculating AUC (area under the curve), sensitivity, specificity, accuracy, positive predictive value (PPV), negative predictive value (NPV), recall, and F-score. The 95% confidence intervals (CIs) for AUC were estimated using DeLong's method. The 95% CIs for sensitivity, specificity, accuracy, false-positive rate, positive predictive value, negative predictive value, Brier score, and F1 score were obtained from 5,000 stratified bootstrap resamples of the held-out validation set. Additionally, SHAP values were estimated using the fastshap package with 100 Monte Carlo simulations based on the predicted probabilities of the corresponding DLM subtype. Mean absolute SHAP values and dependence plots were used to assess feature importance and effect direction. By comparing the SHAP values of different feature factors, one can assess the relative contribution of each feature factor to the model (39). Furthermore, the study will utilize SHAP dependency plots to illustrate the marginal effects of each feature factor on the prediction model's output results, thereby revealing the relationship between the output and the feature variables. Besides, Calibration was assessed in the internal validation set using calibration plots, calibration intercepts, calibration slopes, and Brier scores. Clinical utility was evaluated using decision-curve analysis across threshold probabilities of 0.01–0.50, consistent with the intended use of the models for preliminary risk assessment and referral.

All analyses were performed using R version 4.3.1. The analysis code and detailed model settings are available from the corresponding author upon reasonable request.

## 3 Results

### 3.1 Baseline characteristics

Following assessment of the distributional assumptions for continuous variables, age, height, weight, and BMI were compared among the three groups using one-way analysis of variance or the Kruskal–Wallis test, as appropriate. Gender and meniscus laterality were compared using the chi-square test or Fisher's exact test, as appropriate. No significant differences were observed among the three groups in age, height, weight, BMI, or meniscus laterality (Supplementary Table 1). However, the factor of gender showed significant differences among the three groups (*p* < 0.001). Specifically, complete and incomplete lateral meniscus tears are more prevalent in female patients, with the proportion of female patients being approximately three times that of male patients. This finding is consistent with relevant studies in the existing literature, further supporting the potential role of gender in meniscus morphological differences (3–5).

### 3.2 Imaging parameter analysis

The intraobserver and interobserver ICCs for all radiographic parameters were greater than 0.75 (all *p* < 0.05), demonstrating good measurement reproducibility. The imaging parameters were compared among the three groups using one-way analysis of variance or the Kruskal–Wallis test, as appropriate. The results revealed significant differences in numerous imaging parameters among the three groups (Supplementary Table 2). Further stratified analysis by gender revealed distinct significant imaging parameters among different gender groups. Among males, the primary measurements include the lateral joint space, the tibial eminence width, and the ratio of the tibial eminence width/tibial plateau width (Supplementary Table 3). In the female cohort, the height of the lateral tibial spine, the lateral slope of the lateral tibial spine, and the lateral slope of the medial tibial spine also exhibited statistical significance, in addition to the aforementioned parameters (Supplementary Table 4).

### 3.3 Screening of characteristic variables for the lateral meniscus

The LASSO regression model is utilized for the purpose of screening relevant parameters, with the screening criterion being based on the minimum penalty coefficient λmin. It is evident that at λmin = 0.0092, the model demonstrates optimal fitting performance. Ultimately, six key parameters (gender, lateral joint space, height of lateral tibial spine, lateral slope of the lateral tibial spine, lateral slope of the medial tibial spine, tibial eminence width/tibial plateau width) were selected for subsequent predictive model construction. The distribution of LASSO regression coefficients and the selection process for the optimal penalty coefficient λmin are illustrated in Figure 2.

**Figure 2:**
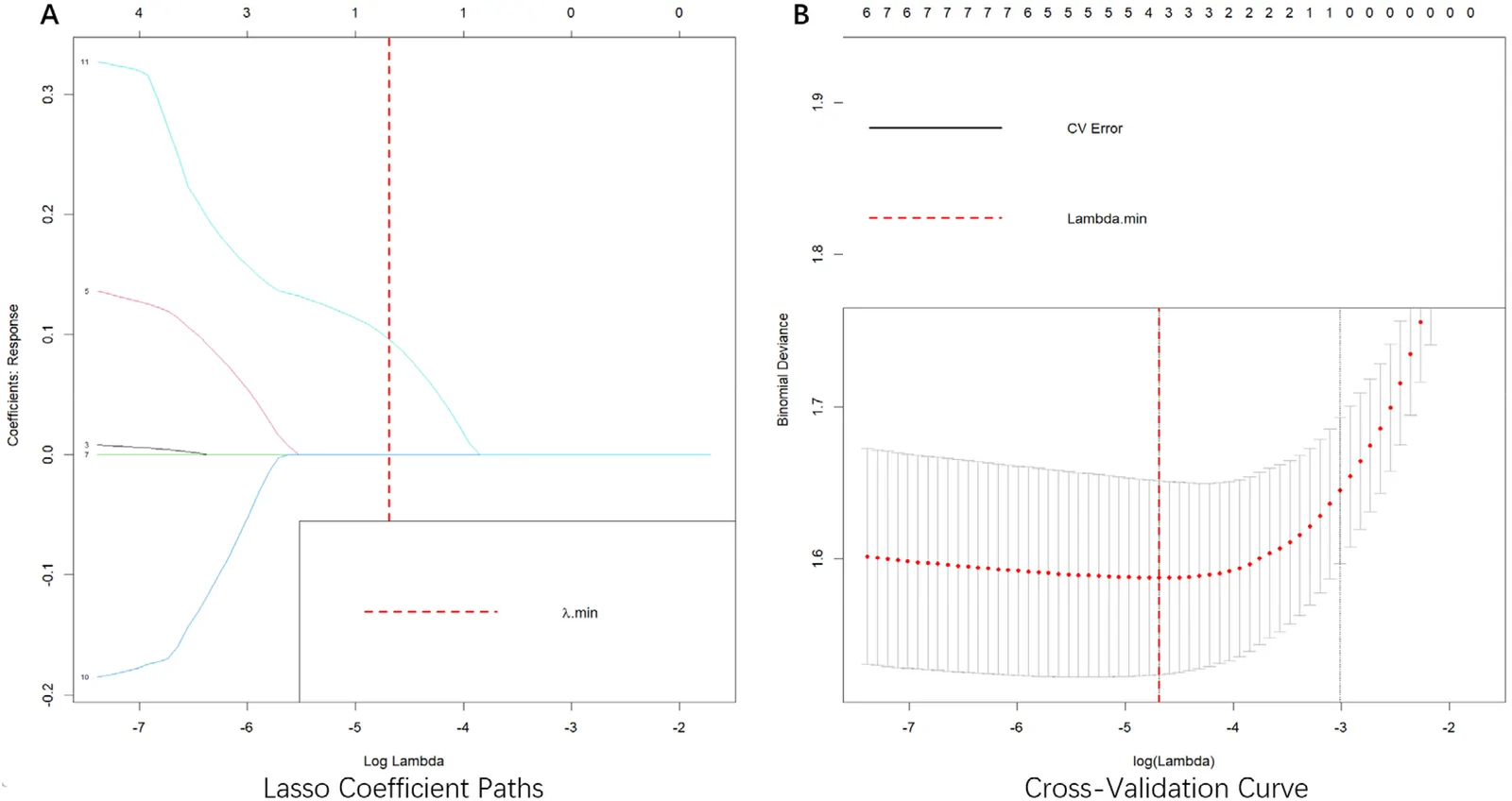
**(A)** Distribution of coefficients for the relevant predictive parameters generated based on the sequence of log(*λ*) values, with the *y*-axis representing the LASSO coefficient distribution for each variable; **(B)** selection of the optimal parameter (*λ*) in the LASSO model. The *λ* value with the best predictive performance in cross-validation is used as the final regularization coefficient.

### 3.4 Performance and factor importance analysis of a clinical predictive model for CDLM

Following model training and hyperparameter optimization in the training set, the performance of each machine-learning model for predicting CDLM was evaluated in the internal validation set. The specific results are shown in Table 1, while the ROC curves and DCA curves for each model are depicted in Supplementary Figures 1, 2.

**Table 1:** Summary of predictive performance indicators of the complete discoid lateral meniscus prediction model.

Predictive Model	AUC	Sensitivity	Specificity	Accuracy	FPR	PPV	NPV	Brier score	F1
Random Forest Model (RF)	0.823 (95% CI: 0.751–0.896)	0.465 (95% CI: 0.326–0.628)	0.941 (95% CI: 0.882–0.988)	0.781 (95% CI: 0.719–0.844)	0.059 (95% CI: 0.012–0.118)	0.801 (95% CI: 0.645–0.952)	0.777 (95% CI: 0.729–0.830)	0.157 (95% CI: 0.133–0.183)	0.588 (95% CI: 0.438–0.720)
XGBoost Model	0.833 (95% CI: 0.752–0.915)	0.674 (95% CI: 0.535–0.814)	0.882 (95% CI: 0.812–0.941)	0.812 (95% CI: 0.742–0.875)	0.118 (95% CI: 0.059–0.188)	0.744 (95% CI: 0.628–0.861)	0.843 (95% CI: 0.787–0.902)	0.152 (95% CI: 0.111–0.198)	0.707 (95% CI: 0.593–0.810)
Support Vector Machine (SVM) model	0.885 (95% CI: 0.829–0.941)	0.535 (95% CI: 0.395–0.674)	0.918 (95% CI: 0.859–0.965)	0.789 (95% CI: 0.727–0.852)	0.082 (95% CI: 0.035–0.141)	0.767 (95% CI: 0.632–0.906)	0.796 (95% CI: 0.745–0.852)	0.147 (95% CI: 0.122–0.171)	0.631 (95% CI: 0.493–0.753)
Logistic Regression Model	0.863 (95% CI: 0.792–0.935)	0.581 (95% CI: 0.442–0.721)	0.941 (95% CI: 0.882–0.988)	0.821 (95% CI: 0.758–0.875)	0.059 (95% CI: 0.012–0.118)	0.833 (95% CI: 0.710–0.958)	0.816 (95% CI: 0.765–0.872)	0.138 (95% CI: 0.110–0.169)	0.685 (95% CI: 0.554–0.795)
K-Nearest Neighbors (KNN) Model	0.826 (95% CI: 0.750–0.903)	0.535 (95% CI: 0.395–0.674)	0.918 (95% CI: 0.859–0.965)	0.789 (95% CI: 0.727–0.852)	0.082 (95% CI: 0.035–0.141)	0.767 (95% CI: 0.629–0.903)	0.796 (95% CI: 0.745–0.849)	0.153 (95% CI: 0.124–0.185)	0.631 (95% CI: 0.492–0.747)
Naive Bayes model	0.855 (95% CI: 0.785–0.925)	0.628 (95% CI: 0.488–0.767)	0.847 (95% CI: 0.776–0.918)	0.773 (95% CI: 0.703–0.844)	0.153 (95% CI: 0.082–0.224)	0.675 (95% CI: 0.564–0.800)	0.818 (95% CI: 0.763–0.878)	0.142 (95% CI: 0.112–0.173)	0.651 (95% CI: 0.537–0.756)
Gradient Boosting Tree Model	0.824 (95% CI: 0.744–0.903)	0.558 (95% CI: 0.419–0.698)	0.941 (95% CI: 0.882–0.988)	0.813 (95% CI: 0.750–0.875)	0.059 (95% CI: 0.012–0.118)	0.828 (95% CI: 0.700–0.955)	0.808 (95% CI: 0.757–0.862)	0.151 (95% CI: 0.123–0.183)	0.667 (95% CI: 0.531–0.781)

In the held-out validation set, all models achieved AUC values greater than 0.80. Among these, the Support Vector Machine (SVM) model achieved the highest AUC value (AUC = 0.885). This model also demonstrated exceptionally high specificity (0.918) and high accuracy (0.789). The DCA curve indicates that the Support Vector Machine (SVM) model demonstrates a higher net gain value, with the remaining metrics also being relatively balanced.

The SHAP values for each predictor in the optimal predictive Support Vector Machine (SVM) model are demonstrated in Figure 3. The findings suggest that lateral joint space was the leading contributor, followed by gender and the remaining anatomical parameters. Higher lateral joint space, female gender, a lower lateral slope of the lateral tibial spine, a higher tibial eminence width-to-tibial plateau width ratio, a lower height of the lateral tibial spine, and a higher lateral slope of the medial tibial spine were associated with a higher predicted probability of CDLM. The SHAP dependency plot (Figure 4) reveals that as lateral joint space increases, female gender, and the lateral slope of the lateral tibial spine decreases, the ratio of the tibial eminence width/tibial plateau width increases, the height of lateral tibial spine decreases, and the lateral slope of the medial tibial spine increases, thereby raising the probability of developing a CDLM.

**Figure 3:**
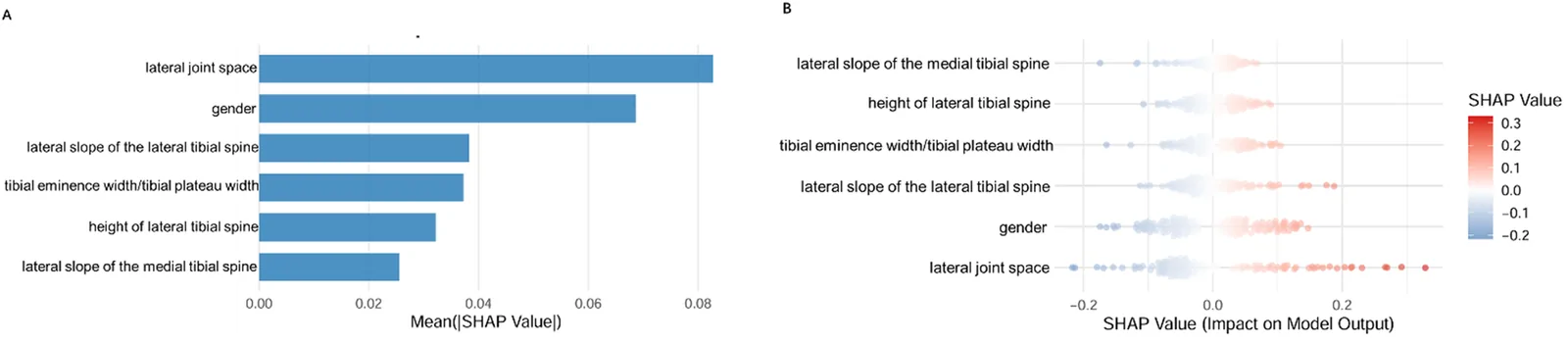
Mean SHAP values of various factors for the complete discoid lateral meniscus based on the support vector machine (SVM) model: **(A)** Bar chart; **(B)** beehive diagram.

**Figure 4:**
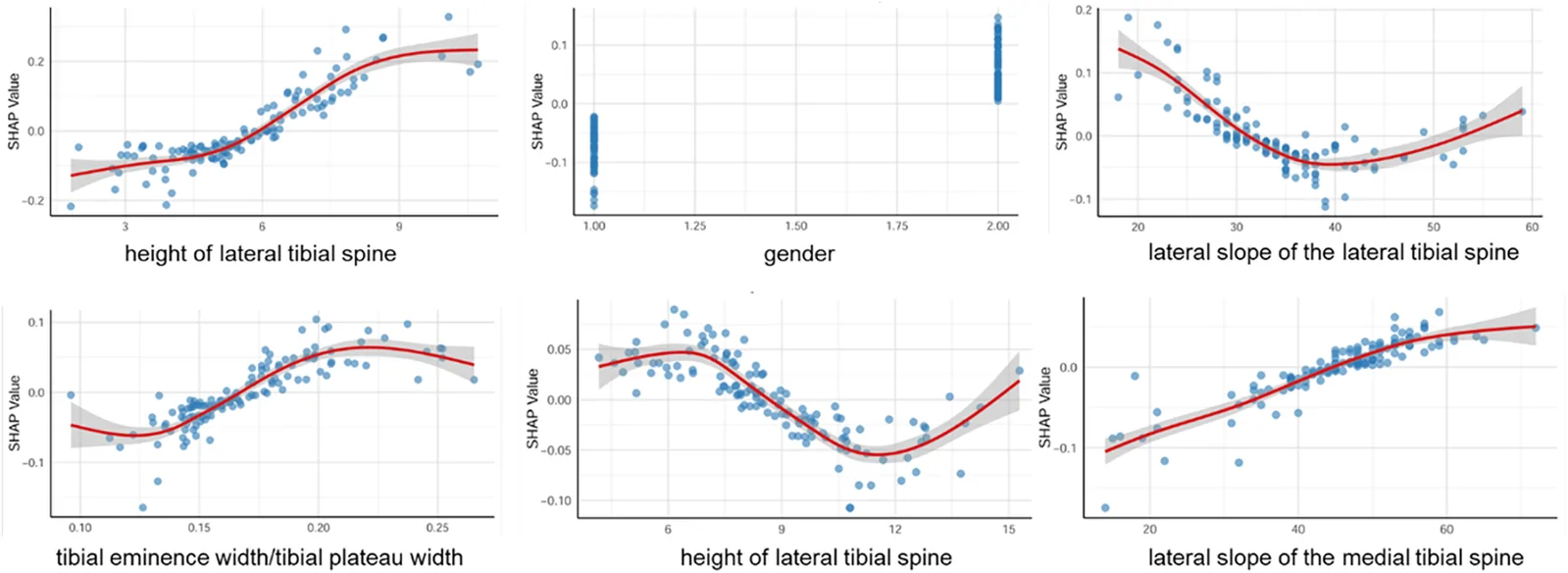
SHAP dependence plots of various factors for the complete discoid lateral meniscus based on the support vector machine (SVM) model.

### 3.5 Performance and factor importance analysis of a clinical predictive model for ICDLM

This study also validated the predictive model's performance in forecasting ICDLM. Following model training and hyperparameter optimization in the training set, the performance of each machine-learning model for predicting ICDLM was evaluated in the internal validation set. The specific results are shown in Table 2. Additionally, the ROC curves and DCA curves for each model are presented in Supplementary Figures 3, 4. The model prediction results indicate that the predictive model based on the aforementioned six parameters demonstrated satisfactory performance in predicting ICDLM. Among these models, the Gradient Boosting model achieved the highest AUC (0.861), while also exhibiting exceptionally high specificity (0.964) and relatively high accuracy (0.856). The DCA curve indicates that the Gradient Boosting model exhibits a relatively high net gain value, with other metrics also showing balanced performance.

**Table 2:** Summary of predictive performance indicators of the incomplete discoid lateral meniscus prediction model.

Predictive Model	AUC	Sensitivity	Specificity	Accuracy	FPR	PPV	NPV	Brier score	F1
Random Forest Model (RF)	0.849 (95% CI: 0.765–0.933)	0.211 (95% CI: 0.053–0.421)	0.976 (95% CI: 0.941–1.000)	0.837 (95% CI: 0.788–0.875)	0.024 (95% CI: 0.000–0.059)	0.667 (95% CI: 0.250–1.000)	0.847 (95% CI: 0.820–0.882)	0.111 (95% CI: 0.090–0.134)	0.321 (95% CI: 0.087–0.538)
XGBoost Model	0.839 (95% CI: 0.757–0.921)	0.369 (95% CI: 0.158–0.579)	0.929 (95% CI: 0.871–0.976)	0.827 (95% CI: 0.769–0.885)	0.071 (95% CI: 0.024–0.129)	0.538 (95% CI: 0.300–0.800)	0.868 (95% CI: 0.831–0.910)	0.131 (95% CI: 0.092–0.173)	0.438 (95% CI: 0.214–0.634)
Support Vector Machine (SVM) model	0.762 (95% CI: 0.646–0.879)	0.321 (95% CI: 0.124–0.518)	0.939 (95% CI: 0.895–0.983)	0.817 (95% CI: 0.766–0.868)	0.061 (95% CI: 0.049–0.137)	0.656 (95% CI: 0.344–0.968)	0.817 (95% CI: 0.782–0.852)	0.139 (95% CI: 0.132–0.146)	0.432 (95% CI: 0.210–0.654)
Logistic Regression Model	0.856 (95% CI: 0.766–0.946)	0.263 (95% CI: 0.053–0.474)	0.976 (95% CI: 0.941–1.000)	0.846 (95% CI: 0.798–0.894)	0.024 (95% CI: 0.000–0.059)	0.714 (95% CI: 0.333–1.000)	0.856 (95% CI: 0.825–0.892)	0.106 (95% CI: 0.083–0.130)	0.385 (95% CI: 0.100–0.600)
K-Nearest Neighbors (KNN) Model	0.715 (95% CI: 0.596–0.834)	0.158 (95% CI: 0.005–0.316)	0.941 (95% CI: 0.882–0.988)	0.798 (95% CI: 0.750–0.846)	0.059 (95% CI: 0.012–0.118)	0.375 (95% CI: 0.002–0.750)	0.833 (95% CI: 0.809–0.863)	0.146 (95% CI: 0.119–0.173)	0.222 (95% CI: 0.012–0.429)
Naive Bayes model	0.685 (95% CI: 0.542–0.827)	0.316 (95% CI: 0.105–0.526)	0.918 (95% CI: 0.859–0.976)	0.808 (95% CI: 0.740–0.865)	0.083 (95% CI: 0.024–0.141)	0.462 (95% CI: 0.222–0.727)	0.857 (95% CI: 0.821–0.898)	0.144 (95% CI: 0.111–0.178)	0.375 (95% CI: 0.154–0.571)
Gradient Boosting Tree Model	0.861 (95% CI: 0.776–0.946)	0.368 (95% CI: 0.158–0.579)	0.964 (95% CI: 0.918–1.000)	0.856 (95% CI: 0.808–0.904)	0.035 (95% CI: 0.002–0.082)	0.701 (95% CI: 0.429–1.000)	0.872 (95% CI: 0.837–0.913)	0.104 (95% CI: 0.076–0.132)	0.483 (95% CI: 0.240–0.688)

The SHAP algorithm was employed to evaluate the importance of six predictive factors. The SHAP values for each predictor variable in the best-performing model (Gradient Boosting model) are shown in Figure 5. SHAP analysis of the Gradient Boosting model identified lateral joint space and the tibial eminence width-to-tibial plateau width ratio as the leading contributors. Higher lateral joint space, a higher tibial eminence width-to-tibial plateau width ratio, a higher lateral slope of the medial tibial spine, a lower height of the lateral tibial spine, and a lower lateral slope of the lateral tibial spine were associated with a higher predicted probability of ICDLM. Female gender showed the same directional association but had limited overall importance. The SHAP dependency plot (Figure 6) reveals that as lateral joint space increases, the ratio of the tibial eminence width/tibial plateau width increases, the lateral slope of the medial tibial spine increases, and the height of lateral tibial spine decreases, female gender, and the lateral slope of the lateral tibial spine decreases, thereby raising the probability of developing a ICDLM.

**Figure 5:**
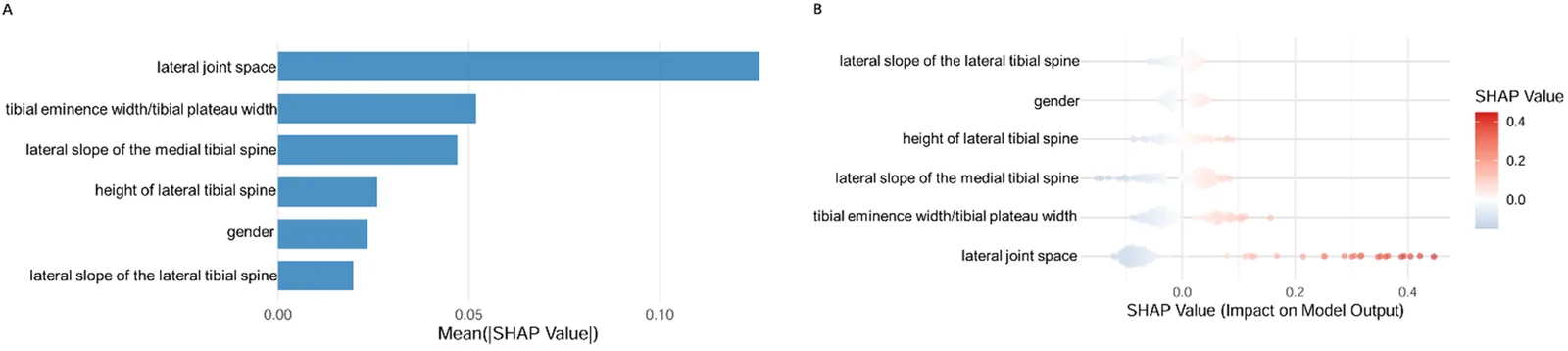
Mean SHAP values of various factors for the incomplete discoid lateral meniscus based on the gradient boosting model: **(A)** Bar chart; **(B)** beehive diagram.

**Figure 6:**
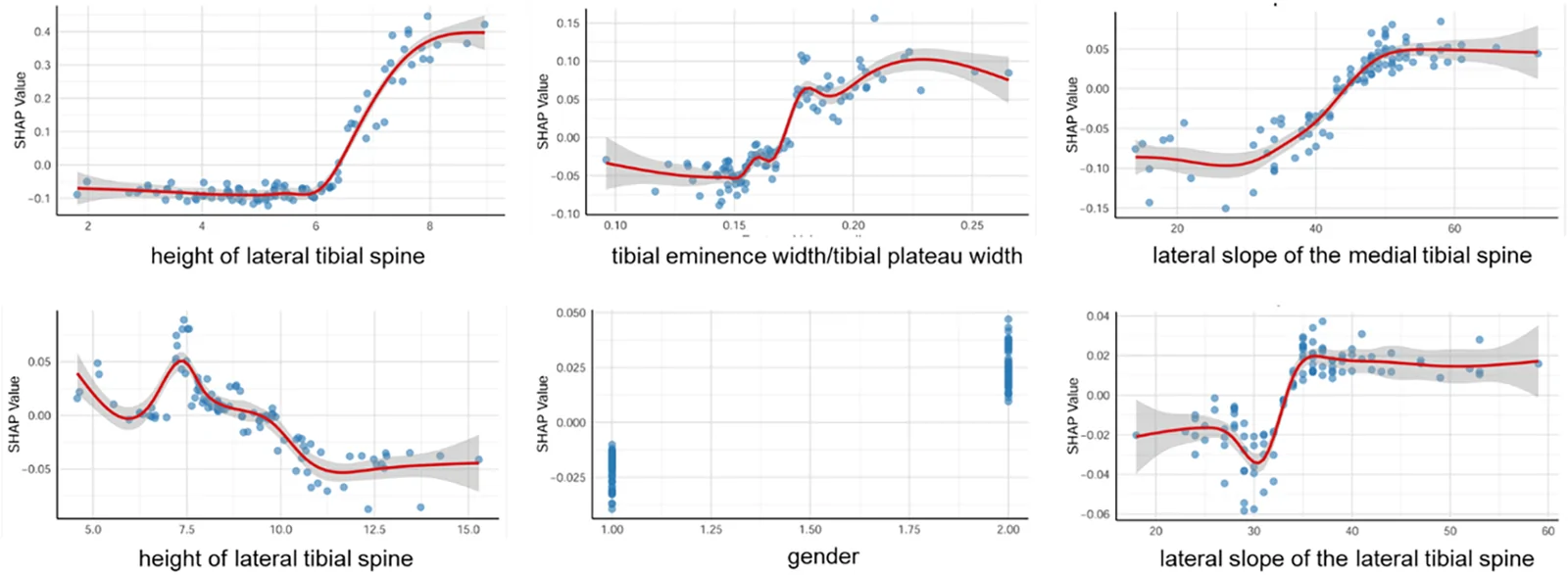
SHAP dependence plots of various factors for the incomplete complete discoid lateral meniscus based on the gradient boosting model.

### 3.6 Calibration and decision-curve analysis

The calibration intercepts/slopes were 0.671/1.848 for the CDLM SVM model and 0.258/1.144 for the ICDLM Gradient Boosting model, with Brier scores of 0.147 and 0.104, respectively. The corresponding calibration plots are presented in Supplementary Figures 5, 6. Decision-curve analysis demonstrated greater net benefit than the treat-all and treat-none strategies at threshold probabilities of 0.13–0.50 for CDLM and 0.02–0.50 for ICDLM, supporting the potential of these models for efficient early screening and risk stratification.

## 4 Discussion

This study used machine-learning models to evaluate the contribution of gender and tibial plateau anatomical parameters derived from routine knee radiographs to the identification of CDLM and ICDLM. The models demonstrated good discriminative performance. SHAP analysis identified lateral joint space as the leading contributor to the SVM-based CDLM model, followed by gender, whereas lateral joint space and the ratio of tibial eminence width to tibial plateau width were the leading contributors to the Gradient Boosting-based ICDLM model. For both subtypes, a wider lateral joint space, a higher tibial eminence width-to-tibial plateau width ratio, a greater lateral slope of the medial tibial spine, a lower height of the lateral tibial spine, and a lower lateral slope of the lateral tibial spine were associated with higher predicted probabilities. Gender contributed more prominently to CDLM prediction but showed limited importance for ICDLM, suggesting that it should be interpreted together with the radiographic parameters rather than as an independent causal determinant. Given their high specificity and comparatively lower sensitivity, these models may serve as accessible adjuncts to routine radiographic assessment by identifying patients with a high predicted likelihood of DLM and supporting timely referral for MRI or specialist evaluation. The calibration and decision-curve findings further support this clinical positioning, while future multicenter prospective validation may help establish broader clinical applicability.

An MRI-based study reported characteristic DLM-associated differences in lateral femoral condylar and lateral tibial plateau morphology, including greater posterior obliquity of the lateral tibial plateau, lateral displacement of the lowest point of the lateral femoral condyle, and a smaller posterior lateral condylar angle (40). The presence of a complete lateral discoid meniscus can be distinguished through radiographic imaging characteristics. Kim et al. conducted a comparative study involving 175 patients and found that patients with complete discoid meniscus had a significantly increased height in the lateral joint space, reaching 6.1 ± 1.9 mm (41). Bellier et al. observed in a follow-up study of 16 children that, despite being in childhood, patients with complete discoid meniscus had significantly higher lateral joint space compared to healthy control children (42). This study also found that as the coverage area of the lateral meniscus on the tibial plateau increased, the height of the lateral joint space increased progressively. Upon completion of the complete transformation of the lateral meniscus into a disc-like structure, the height of the lateral joint space attained its maximum value (6.51 ± 1.49 mm). Moreover, the stratified analysis results from Matthew et al. involving 135 patients indicated that when patients were over 14 years of age, lateral joint space demonstrated higher accuracy in predicting the presence of a lateral discoid meniscus (43). The findings indicate that the development of the lateral compartment of the knee joint is significantly influenced by the morphology of the discoid meniscus, particularly during the period of skeletal growth. This phenomenon may be attributable to the augmented coverage area of the lateral meniscus over the tibia, as well as the heightened demand for lateral space. However, the causal relationship between the lateral joint space and lateral meniscus enlargement remains unclear and requires further validation through long-term follow-up studies.

The height of the fibular head was also among the earliest imaging criteria proposed for distinguishing the discoid lateral meniscus. Kim et al. compared 175 patients and found that the fibular head position in patients with complete discoid lateral meniscus (11.1 ± 3.2 mm) was significantly higher than in those with normal lateral meniscus (14.1 ± 4.0 mm) (41). Furthermore, Song et al. also found that the larger the lateral meniscus area, the higher the position of the fibular head (normal lateral meniscus: 13.6 ± 2.2 mm; incomplete discoid lateral meniscus: 12.6 ± 1.8 mm; complete discoid lateral meniscus: 9.9 ± 2.2 mm) (44). However, following the implementation of a stratified analysis by gender, the parameter of fibular head height no longer exhibited significant differences. Therefore, it can be inferred that the variable of fibular head height is primarily influenced by gender, with a high proportion of female patients with discoid meniscus likely contributing to the reduction in this measurement.

Previous radiographic and MRI studies have identified characteristic differences in lateral femoral condylar morphology in patients with DLM, including the condylar cutoff sign and positional or angular alterations (40, 44, 45). Song et al. compared the development of femoral condyles in 130 patients with discoid lateral meniscus and found that patients with complete discoid lateral meniscus exhibited lateral femoral condyle remodeling with significantly reduced condylar osteotomy landmarks. In contrast, lateral femoral condylar development in patients with ICDLM was closer to normal (44). Ha et al. similarly found that the femoral condyle osteotomy sign serves as a crucial indicator for assessing lateral femoral condyle development, and the femoral condyle osteotomy sign is considered a key marker for lateral femoral condyle remodeling and adaptation to a fully discoid lateral meniscus (45). Additionally, Kinoshita et al. also confirmed through MRI that patients with discoid meniscus exhibit femoral condyle dysplasia (40).

Previous radiographic and MRI studies have indicated that DLM is associated with alterations in tibial plateau and tibial eminence morphology. Park et al. evaluated lateral tibial spine height and lateral tibial plateau obliquity in 119 adults with CDLM and found a significant difference in lateral tibial plateau obliquity, although lateral tibial spine height alone did not differ significantly between the CDLM and control groups (46). Jiang et al. reported significant differences in lateral tibial spine height between patients with DLM and matched controls and proposed 7.8 mm as a radiographic threshold for this parameter. The ratio of lateral joint space distance to lateral tibial spine height also demonstrated diagnostic value, with a sensitivity of 73.6%, a specificity of 83.0%, and an AUC of 0.80 (47). Using MRI, You et al. assessed the heights of the medial and lateral tibial spines, tibial eminence width and its ratio to tibial width, and the lateral slopes of the medial and lateral tibial spines. The lateral slope of the medial tibial spine was smaller in the pediatric DLM group, whereas the differences in medial and lateral tibial spine height, tibial eminence width, and lateral slope of the lateral tibial spine were not statistically significant (48). In the present study, SHAP analysis showed that a lower height and lateral slope of the lateral tibial spine, a higher lateral slope of the medial tibial spine, and a higher tibial eminence width-to-tibial plateau width ratio were associated with higher predicted probabilities of both CDLM and ICDLM. The difference in the direction of the medial tibial spine slope compared with previous MRI findings may be related to differences in patient age, imaging modality, measurement definitions, or DLM subtype. Collectively, these findings suggest that DLM is associated with coordinated morphological changes in the tibial eminence rather than an isolated osseous parameter. Increased lateral meniscal coverage may alter the mechanical environment of the tibial plateau during skeletal development, potentially contributing to flattening of the lateral tibial spine and compensatory remodeling of the medial tibial spine; however, this developmental mechanism remains to be confirmed in longitudinal studies.

This study still had some limitations. This study was conducted at a single center, and model performance was assessed through internal validation. Independent multicenter and prospective validation is therefore warranted to confirm its applicability across different patient populations, imaging protocols, and clinical settings. Furthermore, the effects of model-assisted assessment on clinical workflow, preventive management, and patient outcomes have not yet been prospectively evaluated.

## 5 Conclusion

This study developed and internally evaluated seven machine-learning models using gender and five routinely obtainable radiographic parameters: lateral joint space, lateral slope of the lateral tibial spine, height of the lateral tibial spine, lateral slope of the medial tibial spine, and the ratio of tibial eminence width to tibial plateau width. Among the evaluated algorithms, the SVM and Gradient Boosting models demonstrated the best discriminative performance for CDLM and ICDLM, respectively. The calibration and decision-curve analyses further supported the potential clinical relevance of the model-derived risk estimates. By integrating these anatomical parameters, the models may provide an accessible radiograph-based adjunct for identifying patients with a high predicted likelihood of DLM, thereby supporting early recognition and timely referral for MRI or specialist evaluation. Future multicenter prospective studies are warranted to confirm their generalizability and clinical utility.

## Data Availability

The raw data supporting the conclusions of this article will be made available by the authors, without undue reservation.
