# Supplementary material for: Prediction of discoid lateral meniscus based on tibial plateau anatomical factors and machine learning
Source: Front Med (Lausanne). 2026 Sep 28;13:1814295. doi: 10.3389/fmed.2026.1814295 (PMC13654740; doi:10.3389/fmed.2026.1814295)
Supplement: Supplementary file 1 [file Datasheet1.pdf]

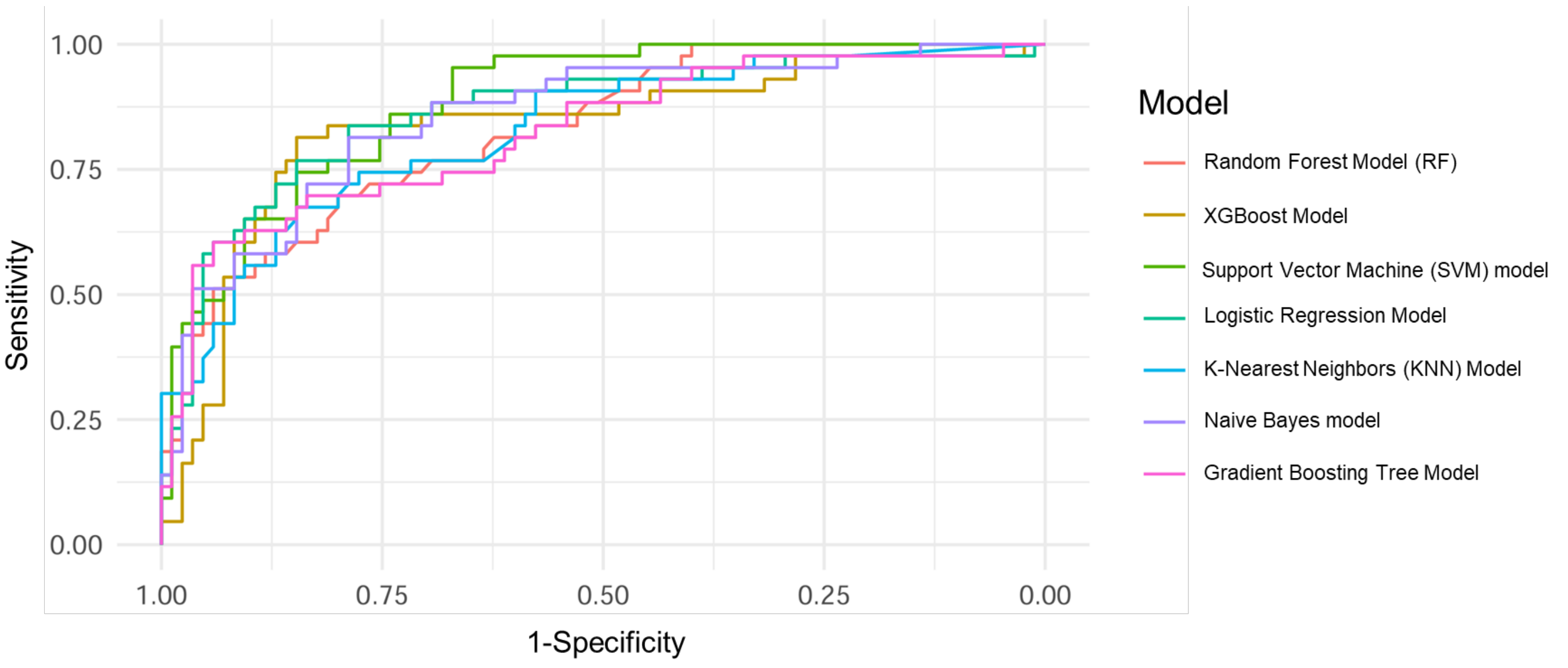

Supplementary Figure 1： ROC curve of the predictive model for complete discoid lateral meniscus

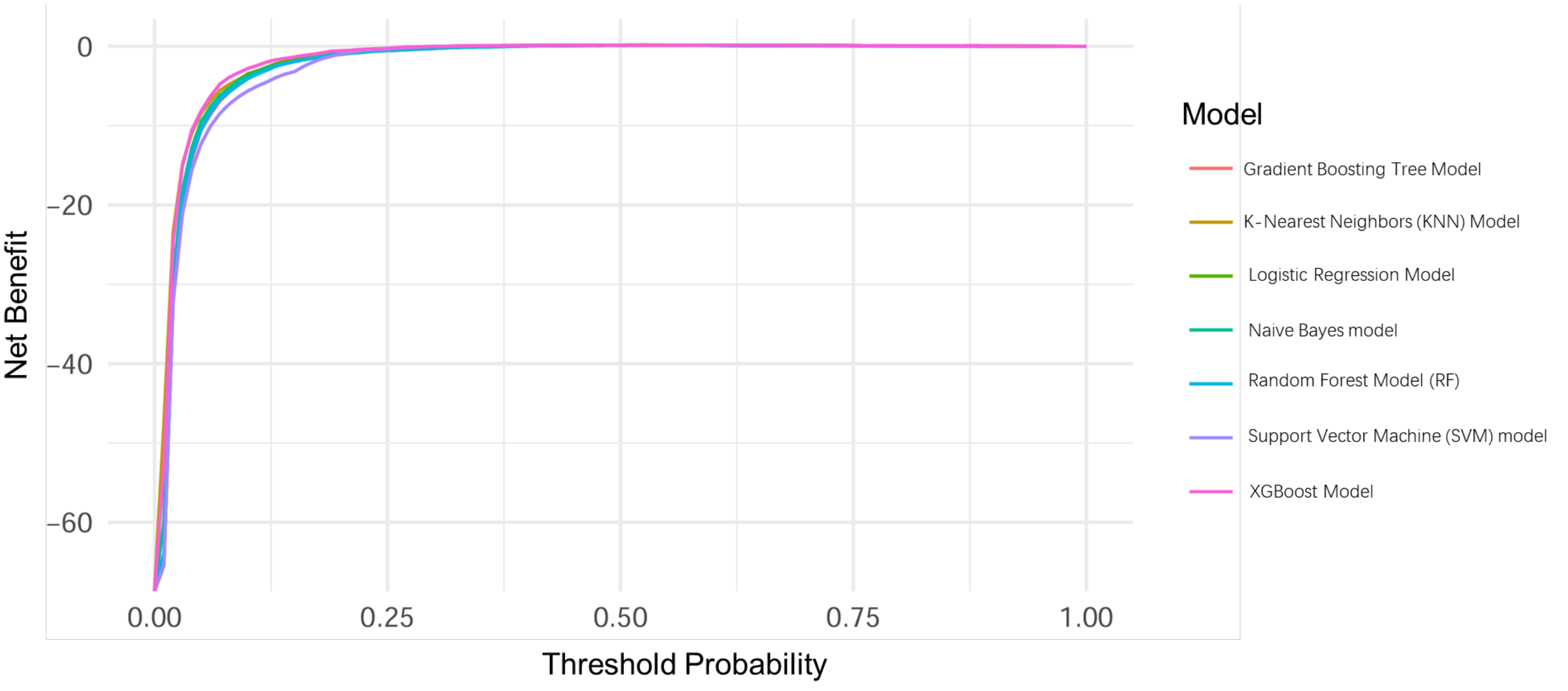

Supplementary Figure 2： DCA curve of the predictive model for complete discoid lateral meniscus

朴素贝叶斯（Naive Bayes）模型

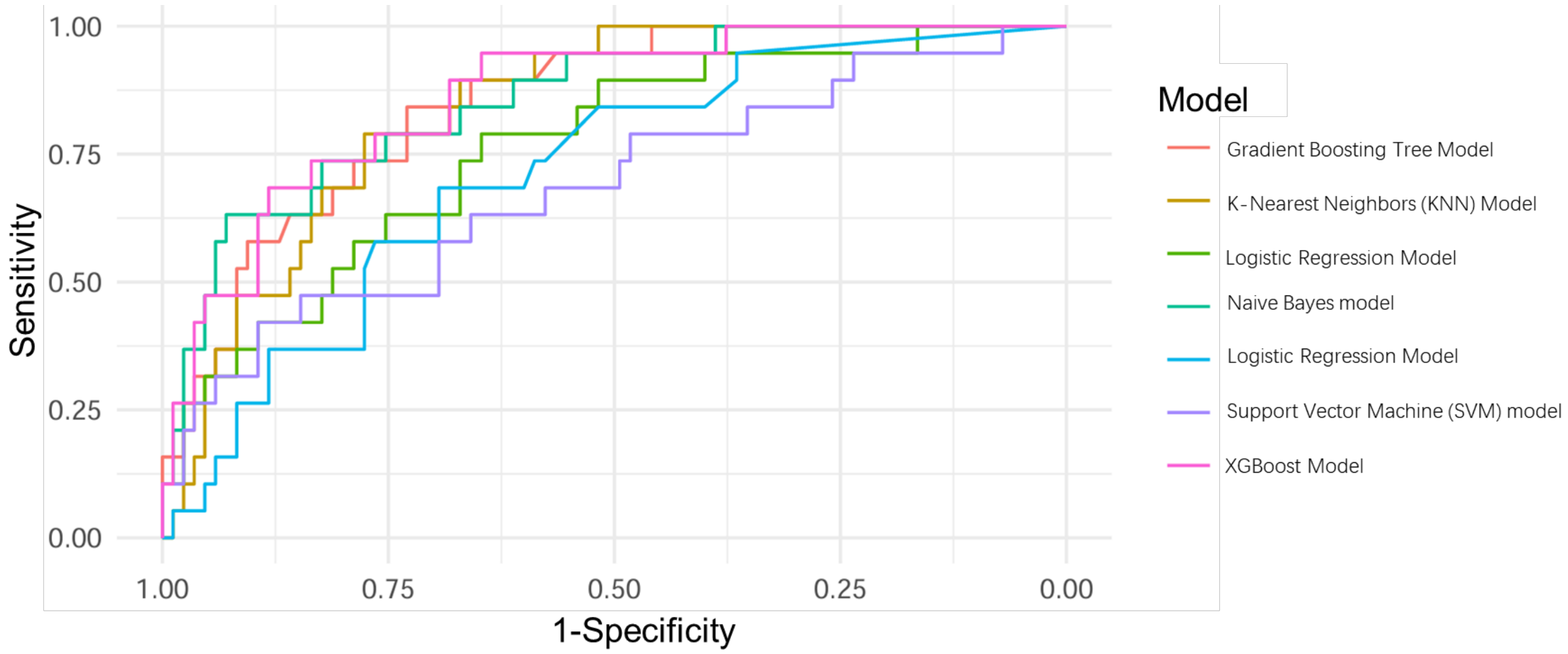

Supplementary Figure 3: ROC curve of the predictive model for incomplete discoid lateral meniscus

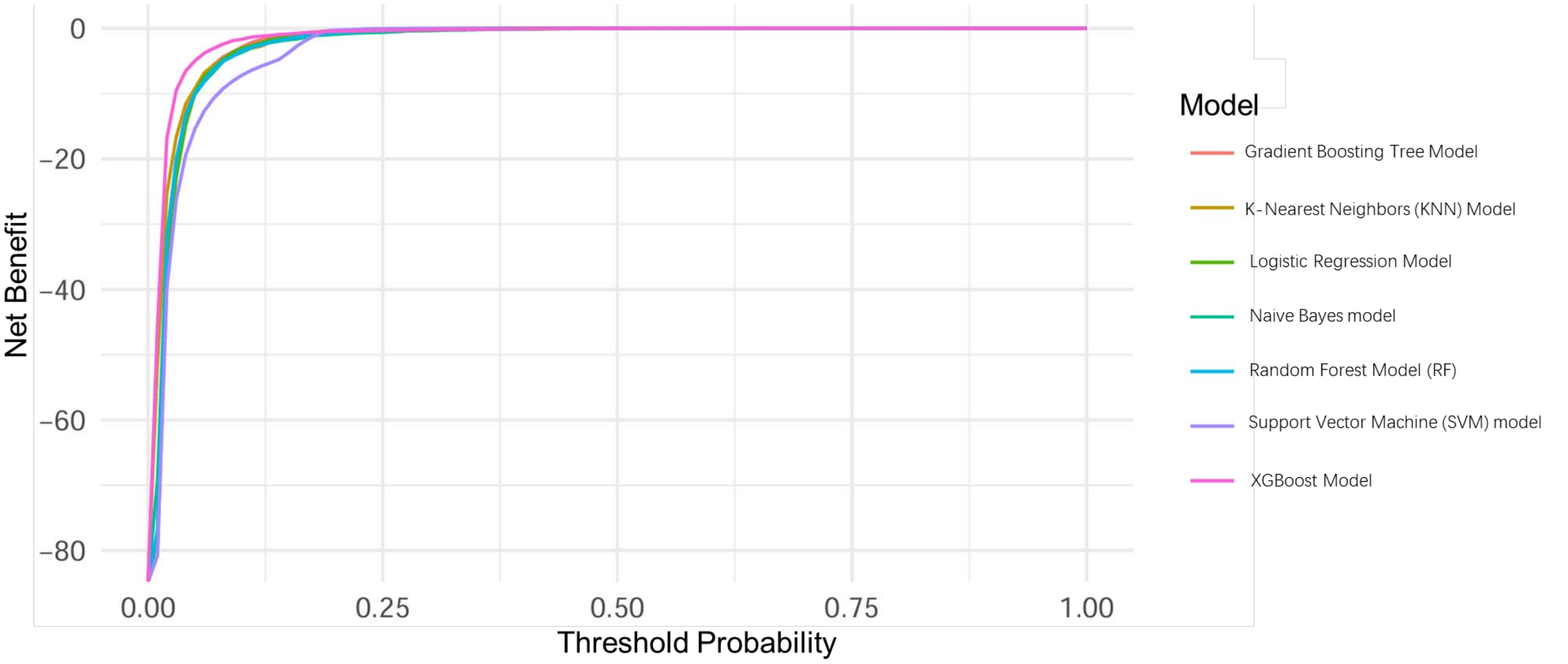

Supplementary Figure 4: DCA curve of the predictive model for incomplete discoid lateral meniscus

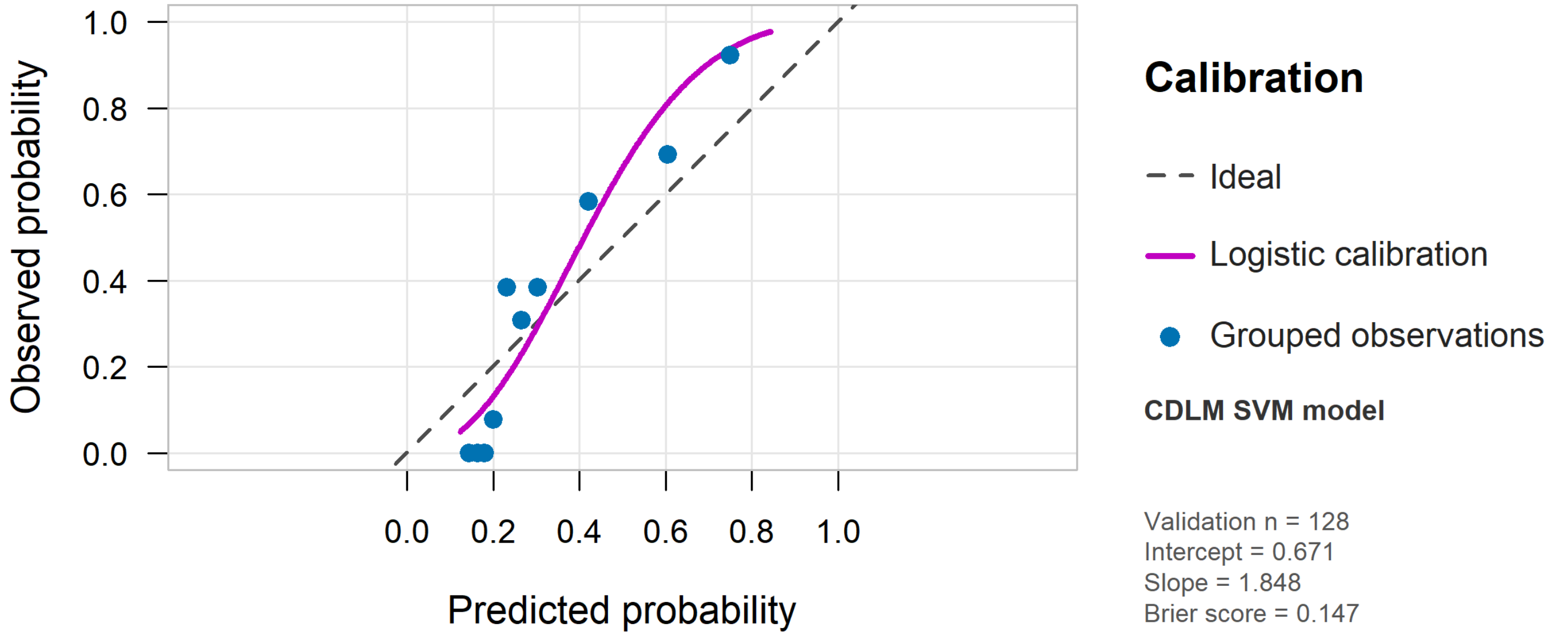

Supplementary Figure 5. Calibration plot of the final support vector machine model for complete discoid lateral meniscus in the internal validation set.

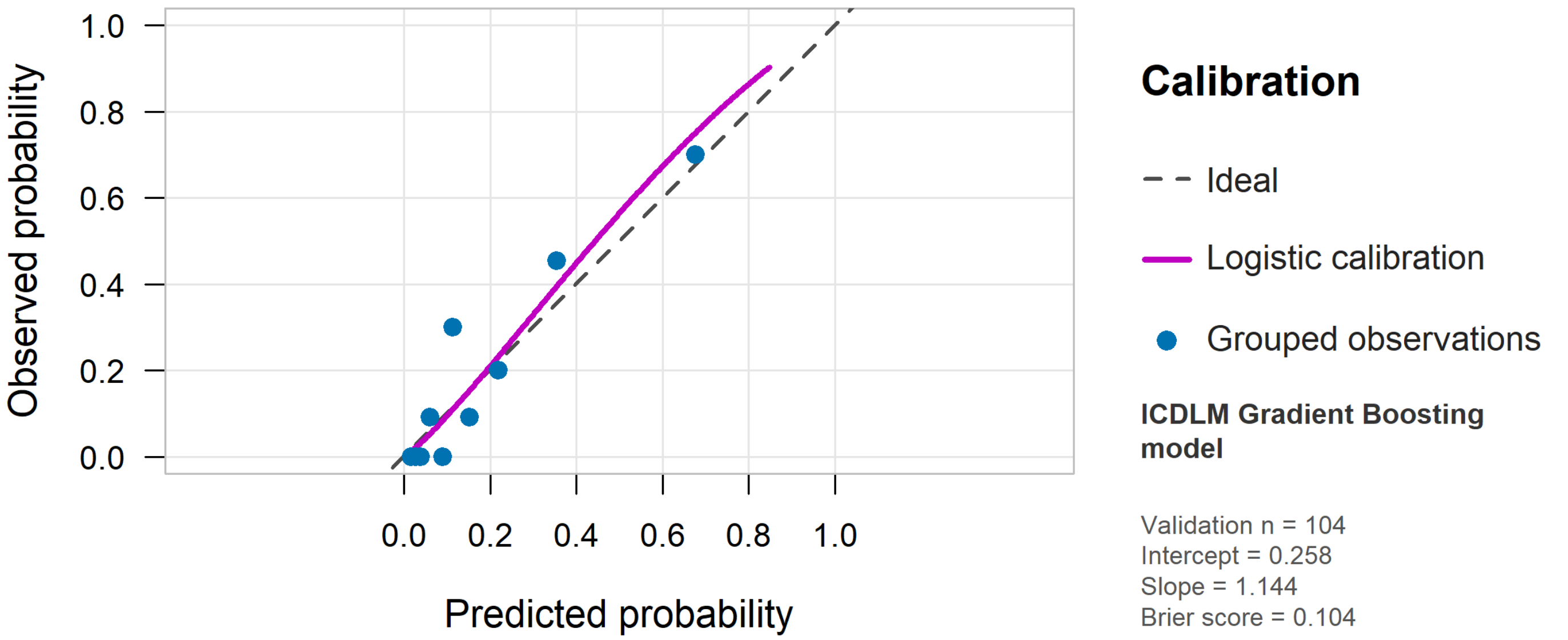

Supplementary Figure 6. Calibration plot of the final Gradient Boosting model for incomplete discoid lateral meniscus in the internal validation set.
